# Twilight Sleep

**Published:** 1916-06

**Authors:** J. B. Dorrell

**Affiliations:** Springfield, Mo.


### Twilight Sleep.

J. B. DORRELL, M. D., SPRINGFIELD, MO.

Human progression is not unlike the course of the Mississippi river which rolls along and over in its bed, changing its location at will, without regard for the feelings or the interests of those who think they own the properties along its banks. So it demolishes a valuable plantation here, and leaves a bar across the channel, and the neighbor may make the most of it. Metternich closed the book after the fall of Napoleon, crossed his arms, and said: "That ends it. The world is settled forever. No more war." And just look at Europe now! Long before his time that same folding of the hands in peace had been seen in the days of Diocletian, Alexander, Severus, Augustus, Solomon, Darius, and other wise rulers. But no sooner were affairs settled than they became again topsy-turvy, as at present.

Now that we have gotten rid of the great menace of slavery, have conquered the West and banded the land with rails, curbed the trusts and made Standard Oil pay its taxes, we might reasonably look for a period of repose—when along comes Ruth, and says she wants her rights. The disquieting phenomenon of the day is this uprising of woman. She wants to vote; she wants to select the right sort of a father for her weans; she wants freedom and equality; she wants so much that she finds difficulty in stating it comprehensively. Moreover, all of us who are wedded know that when she really wants anything it is a mere question of time until she gets it.

This feminine unrest threatens to crystallize about the question of painless childbirth. The better half of our union has declared our half unfair. She says: "The day has passed when the women did the work and the men did the fighting. You don't need to fight, and we cannot afford to have the best built men killed off. Even as it is, many of us have to put up with pretty scrubby specimens or go without. We have all the pain and the peril to encounter, while you go gayly off about your politics or your business and leave us stretched on the bed of anguish to bear the children of both. We have put up with this because we had to; but if we don't have to, we won't; so there!"

It is by no means a trivial or non-significant thing that womankind has taken up this twilight sleep as she has. Read the publications like that of Marguerite Trace and Mary Boyd, and you must see that the demand is one that must be satisfied. The women say: "We are not pleading for scopomorphine, or for the Freiburg method; we demand painless childbirth; and if this is not the best variety, produce the better."

It is no use to get huffed, and assert the rights of our profession to decide such questions; if we refuse her demand, she will go to another doctor who has more complaisance—or more humanity. The fact is simply this—we always oppose any radical improvement in our own line, and only accept it when it is forced upon us; then we get very enthusiastic and proclaim it as an evidence of the vast advances *we* are making. Oh, we are fine fellows, only we get into very deep ruts and do not like to be jarred out of them. But real hard jarring is good for the soul, and without it we would be content to go along from day to day, making our rounds delivering a baby here, soothing a deathbed there, and collecting enough to keep the wolf from our door.

Let us agree that the demand of the women is just, and that it is up to us to remove the curse from child-bearing as far as we are able to do so with safety to mother and child. The question then turns to the better method to be employed.

Having considered the conservatism of our profession, we come here upon another cardinal trait, its independence; and just as some fastidious carpers may denominate our conservatismruttiness, so they may term our independence jealousy. Be that as it may, it is certain that we do not like to accept other doctors' ideas, but always like to substitute others of our own, as at least just as good. The original method as introduced by Kroenig and Gauss has scarcely received a cursory trial here, but a whole flock of modifications and substitutes have taken its place. We have spinal anesthesia, gas-oxygen, gas-ether, and others; but for the Freiburg method we have several level-dose methods. The Gauss plan involves a first dose of morphine and scopolamine, followed by the latter alone, the morphine being repeated only in exceptional instances. Meanwhile every possible accessory is employed to favor the analgesia, and lessen the quantity of drug to be used to the lowest possible point. Gauss' first dose consists of one-fourth grain morphine and from 1-150 to 1-30 grain of scopolamine. Subsequent doses are much smaller of scopolamine, and contain no morphine. The intervals vary according to the memory test from one and one-half to four hours.

Gauss thus describes the progress of the amnesia: The first sign of the drug action is a sense of weariness, soon passing into a peaceful sleep, occupying the intervals between pains, which are felt but less acutely, or as not painful. Then follow thirst, dryness of the mouth and throat, face flushing, sometimes slight twitching of the finger flexors, or some motor restlessness. With adequate doses the sleep becomes deeper, the patient does not become fully awake during pains, which are noted by slight facial contortions and groans. Consciousness is retained, and the patient can remember anything done to her. Many stop here; Gauss pushes the drug further. A little deepening of the semi-narcosis suffices to cloud consciousness without abolishing it, so that the condition is reached where the amnesia is complete for the whole period of childbirth.

Unless this stage is induced the patient will think she suffered even though she did not; and one of the principal benefits of the method is lost. The condition is thus described by Gauss: "Most patients seem sleepy but otherwise quite normal. Every pain is accompanied by some expression of suffering. The pains are referred to as such; every question is answered, sleepily but clearly." Yet this apparently conscious patient after the birth has not the slightest idea of what she has been through or what has been said to or by her. It is easy to see that a casual visitor physician though he may be, hearing the woman's expressions of suffering, may conclude that the method is a failure.

The results may be quickly summed, without resorting to tedious and often misleading statistics. The mortality at the Freiburg Frauenklinik was cut in two by the introduction of the Daemmerschlaf, or from 3.4 per cent of the babies born there to 1.3 per cent. Also this klinik has the lowest death rate for mothers and infants of any in Europe. Great as is the direct and immediate benefit conferred by this method, Gauss himself asserts that it is less important than the prevention of the fear of subsequent maternity to the mother, and the prevention of mental and nervous disease that is often induced by the suffering and the terrors of child-bearing.

As to the safety of the method, Hatcher, in 1911, acknowledged that no deaths had been proved in obstetric cases against the combination of morphine and scopolamine. That people die is a well known fact; and that we are not always able to give the reason for demise is also acknowledged; but when they die from the action of a poison we should be able to detect this, and to tell how it acts and why the death follows it. Otherwise all we can truthfully say is that the patient received such and such a medicine and died; but the connection between the two is not closer than that of the ancient beldame who, hearing that a friend had died while sipping a cup of tea, thereafter foreswore her tea. Surely the day has come when we can demand something like certainty in the study of lethal cases of poisoning.

One of the most thorough and careful reviews of the subject that has yet appeared in America is a volume by Hellman, of the Lebanon and German hospitals of New York City. His review is followed by reports of sixty-six cases. His conclusions are: "Under proper surroundings and under proper conditions and in properly selected cases this treatment is ideal. Conducted along the lines laid down by Gauss, it is absolutely safe for mother and child."

Studying the reports from Germany, one cannot avoid the conviction that most of the opposition there came from the fact that a very small university, of Baden, announced a great discovery which was not relished by the very great University of Berlin; and most of Europe as well as America has been dominated by Berlin. Wherever the method elaborated by Gauss has been applied the reports are favorable; when other methods are employed the results reported are not nearly so good.

The principal objection is that the method requires close attention on the part of the attending physician, if this can be called an objection. The memory test demands this. Any occurrence may be taken, as for example, the use of a watch, ask time; if in ten minutes after this the patient is asked the time again and remembers it, she needs another dose of scopolamine. But it surely seems that the woman is entitled to at least this much attention from the one she has chosen to care for her during this supreme ordeal of her life.

---

Dr. and Mrs. J. H. Brice, of Knox City, have moved to Lamesa, where Dr. Brice has just built a fine sanitarium.

---

Said the bibulous gentleman who had been reading birth and death statistics: "Do you know, James, every time I breathe a man dies?"

"Then," said James, "why don't you chew cloves?"

---

"Of course, doctor, German measles are seldom serious?"

"I never met but one fatal case."

"Fatal?"

"Yes; it was a Frenchman, and when he discovered that it was German measles that he had, mortification set in."—*Holland's.*

